# IUC26600-79 ‘State-of-the-Nation’ Study: Understanding the current NHS treatment pathway to identify opportunities to advance future care of patients with high-risk non-muscle invasive bladder cancer in the UK (SPAN-UK)

**DOI:** 10.1093/oncolo/oyag286.006

**Published:** 2026-08-21

**Authors:** J Aning, J Catto, R Martin, K Chatterton, P Mariappan, S Mccormack, S Gill, B Szabados

**Affiliations:** Bristol Urological Institute, North Bristol NHS Trust, Bristol, UK - United Kingdom; Division of Clinical Medicine, School of Medicine & Population Health, University of Sheffield, UK - United Kingdom; Royal Marsden NHS Foundation Trust, London, UK - United Kingdom; Guy’s and St Thomas’ NHS Foundation Trust, London, UK - United Kingdom; Edinburgh Bladder Cancer Surgery (EBCS), University of Edinburgh / Western General Hospital, Edinburgh, UK - United Kingdom; Johnson & Johnson Innovative Medicine - United Kingdom; Johnson & Johnson, Innovative Medicine - United Kingdom; University College London Hospitals NHS Foundation Trust, London, UK - United Kingdom

## Abstract

**Background:**

Optimal outcomes in high-risk non-muscle-invasive bladder cancer (HR-NMIBC) depend on coordinated, adequately resourced Multidisciplinary Team (MDT) care and robust service-level quality performance indicators (QPIs). To date, the HR-NMIBC care pathway across all National Health Service (NHS) regions has not been mapped. This descriptive, cross-sectional study was therefore undertaken to characterise the evolving treatment pathway from diagnosis through to follow-up, identifying areas for optimization, understanding MDT structure and current decision-making.

**Methods:**

A survey methodology was developed with a UK clinical steering group, ensuring relevance. Between June and October 2025, structured online interviews were completed by 70 Health Care Professionals (HCPs) across the UK. The secure electronic survey platform captured quantitative and qualitative data with national representation across institutions and roles. Quantitative items were summarised descriptively; free-text responses underwent thematic analysis.

**Results:**

Referrals from primary care mostly entered via haematuria/Two-Week-Wait clinics. Overall, 87% reported diagnosis communication within ≤6–8 weeks of referral, while 11% reported delays of > 8 weeks. 70% reported insufficient specialist bladder cancer nurse resources. Bacillus Calmette–Guérin (BCG) was typically utilised as adjuvant therapy (98.6%) for HR-NMIBC; however, maintenance duration varied (median 20% completed ≥2 years; 60% ≤1 year). Real-world BCG challenges reportedly included toxicities (87.1%), limited options after BCG (77.1%), and recurrence on/after BCG (57.1%). In BCG-unresponsive disease, approximately 55% of patients tend to be eligible for and consent to radical cystectomy (RC), whereas the remaining 45% tend to be either ineligible or decline despite eligibility. Reasons for declining RC tend to include patient preference for bladder preservation, concerns about morbidity/mortality, and quality-of-life concerns. Trial interest was high, but access was inconsistent. All respondents endorsed adherence to QPIs and the need for national auditing in HR-NMIBC.

**Conclusions:**

This UK-wide assessment highlights an ongoing need to optimise the HR-NMIBC pathway. Priorities include enhancing specialist nurse resourcing; streamlining diagnostic and treatment pathways; standardising BCG protocols plus systematically tracking outcomes; expanding access to evidence-based bladder-sparing treatments and to clinical trials. Addressing these areas may reduce regional variation and lower progression risk.

